# Microalgal hydrogen production: prospects of an essential technology for a clean and sustainable energy economy

**DOI:** 10.1007/s11120-017-0350-6

**Published:** 2017-02-26

**Authors:** Vinzenz Bayro-Kaiser, Nathan Nelson

**Affiliations:** Department of Biochemistry and Molecular Biology, The George S. Wise Faculty of Life Sciences, Tel Aviv University, 69978 Tel Aviv, Israel

**Keywords:** Microalgae, Photosynthetic hydrogen, Sustainability, Energy

## Abstract

Modern energy production is required to undergo a dramatic transformation. It will have to replace fossil fuel use by a sustainable and clean energy economy while meeting the growing world energy needs. This review analyzes the current energy sector, available energy sources, and energy conversion technologies. Solar energy is the only energy source with the potential to fully replace fossil fuels, and hydrogen is a crucial energy carrier for ensuring energy availability across the globe. The importance of photosynthetic hydrogen production for a solar-powered hydrogen economy is highlighted and the development and potential of this technology are discussed. Much successful research for improved photosynthetic hydrogen production under laboratory conditions has been reported, and attempts are underway to develop upscale systems. We suggest that a process of integrating these achievements into one system to strive for efficient sustainable energy conversion is already justified. Pursuing this goal may lead to a mature technology for industrial deployment.

## Introduction

### Global warming—a severe global problem requiring a global solution

Global warming is probably the most challenging problem that humanity faces. It is caused by air pollution and is triggering an increase in average global temperatures. The increase in temperature has had and will continue to have a dramatic effect on society and economy. The major concern is its effect on agriculture and global food security (Wheeler and von Braun 2013; Tai et al. 2014). This effect is expected to be especially powerful in regions facing already harsh climate conditions, exacerbating poverty and hunger. The consequences can include mass migration, social and political instability, and even military conflicts. Other related major issues are rising sea levels, ocean acidification, and increased mortality due to air pollution (Schaeffer et al. 2012; Kroeker et al. 2013; IEA 2016). The major contributor to air pollution is the use of fossil fuels for energy production (IEA 2015a). There is an acute shortage of relatively clean energy, which is required to replace fossil fuels and meet the increase in energy demand to ensure energy security. Air pollution and climate change affect the entire world and require a clean energy solution at a global scale.

Since the industrial revolution, humanity has been emitting greenhouse gases (GHGs) into the atmosphere in great disproportion to their assimilation. This ongoing shift in the atmosphere results in climate change, which is happening much faster than any possible evolutionary adaptation process. This brings our planet out of balance and established ecosystems and agriculture are endangered (Corlett and Westcott 2013; Moritz and Agudo 2013; Franks et al. 2014). In view of this threat to our future, leaders of most countries agreed on combining efforts to reduce GHG emissions and limit average global warming to 2 °C over pre-industrial average global temperatures (UN FCCC-Conference of the Parties (COP) 2015). Attaining this goal would prevent the most dramatic consequences of global warming but would still entail great economic and social damage. Given the current technological and political environment, a more ambitious goal seems out of reach. However, the world has to update and increase the targets in the future to reduce the damage of climate change even further. To have a 50% chance of achieving the 2 °C goal, a maximal amount of anthropogenic GHG emissions are allowed. The major GHG is CO_2_, and its allowed anthropogenic emission budget is about 3000 Gigatonne (IEA 2015a). Almost 2000 Gigatonne is estimated to have been emitted already by the year 2014. If the world continues with the current trend of increasing CO_2_ emissions every year, the remaining emission budget will be consumed by the year 2040. Instead, to reach the set goal, a peak in CO_2_ emissions needs to be reached by the year 2020 and then steadily reduced to zero or almost zero anthropogenic emissions. Meanwhile, our ever-growing world population is expected to increase by 33% by 2050. Simultaneously, there is an expanding worldwide effort to establish energy security and food security and eradicate poverty by economic growth. Reducing global GHG emissions without compromising economic growth will be a tremendous challenge that will require altering policies driven by national interest towards policies for a global solution. The International Energy Agency (IEA) assessed that with the current available technology and policies, the 2 °C goal cannot be achieved, but they point out viable measures to delay the consumption of our GHG budget. Implementing the proposed measures would place the world in a “bridge scenario” which would gain some time. However, to achieve the 2 °C goal, development and deployment of new technologies on a global scale will be necessary.

### Energy sector—the main GHG contributor requires a complete transformation

The energy sector is the major contributor to human GHG emissions. It is dominated by fossil fuels, and their use accounts for 66% of total GHG emissions (IEA 2015a). Coal is the most abundant and cheap fossil fuel and the most polluting one. The gravity of coal use can be observed by the air pollution in Beijing or Shanghai. Fossil fuels are carbon-rich energy carriers and a product of two transformation steps. The first is photosynthesis, which consists of using solar energy and water for CO_2_ fixation into carbon-rich organic compounds. The second is deoxygenation of organic matter, a process that takes millions of years at high temperature and pressure under the earth’s crust (Sato 1990). Fossil fuels have a high energy density and are very flexible energy carriers. Not much effort is required for their extraction, and they can be transported worldwide before being easily transformed into different types of energy that humans require. Additionally, they can be stored and used on board isolated systems like vehicles. These great advantages made fossil fuels the motor of the industrial revolution, enabled high technology development, and sustain our modern lifestyle. In 2013, fossil fuels covered 82% of the total 18 TWyear of the global energy demand (IEA 2015e). However, in the last few decades, it has become clear that the rate by which humans consume fossil fuels cannot be compensated by the rate of photosynthetic CO_2_ fixation and new fossil fuel generation. Thus, fossil fuels are not consumed as a recyclable energy carrier. Instead, they are exploited as a primary source of energy. The continued use of fossil fuels at current rates would result in the undesired transformation of our atmosphere and eventual depletion of our main source of energy. In view of this, a complete transformation of the energy sector to a relatively clean and sustainable system will be required to limit global warming and ensure sustained energy security. Nuclear and hydro energy are currently the major GHG emission-free energy sources. However, they will not have the capacity to replace fossil fuels and cover the increasing energy demand.

Nuclear energy is a highly deployed technology with around 400 operating reactors worldwide. They covered 1.5% of global energy demand in 2013 (IEA 2015b). Further deployment is of great interest to reduce GHG emissions. In 2015, ten reactors were connected to the grid, adding a capacity of 9 GW, and the construction of seven new reactors was begun. Taking into account the current capacity factors, 263 reactors need to be connected to the grid each year until 2050 to cover 50% of the energy demand covered by fossil fuels in 2013. Ramping up the deployment to such a scale would magnify the problems related to nuclear energy (e.g., radioactive waste disposal, plant decommissioning, civil safety) to an unacceptable level.

In contrast to nuclear energy, hydro energy is clean and sustainable. Solar energy is naturally converted into gravitational potential energy by water evaporation, and the resulting freshwater runoff is used to generate electricity. In 2013, hydro energy generated 0.42 TWyear, covering 2.4% of the global energy demand (IEA 2015e). The theoretical global potential derived from the total runoff is estimated at 4.8 TWyear, which entails a technical potential of 1.7 TWyear (IEA 2012). Developing the remaining technical potential would cover only 8.7% of the energy demand met by fossil fuels in 2013. Creating artificial seawater runoff might be considered to increase the potential of hydro energy.

**Fig. 1:**
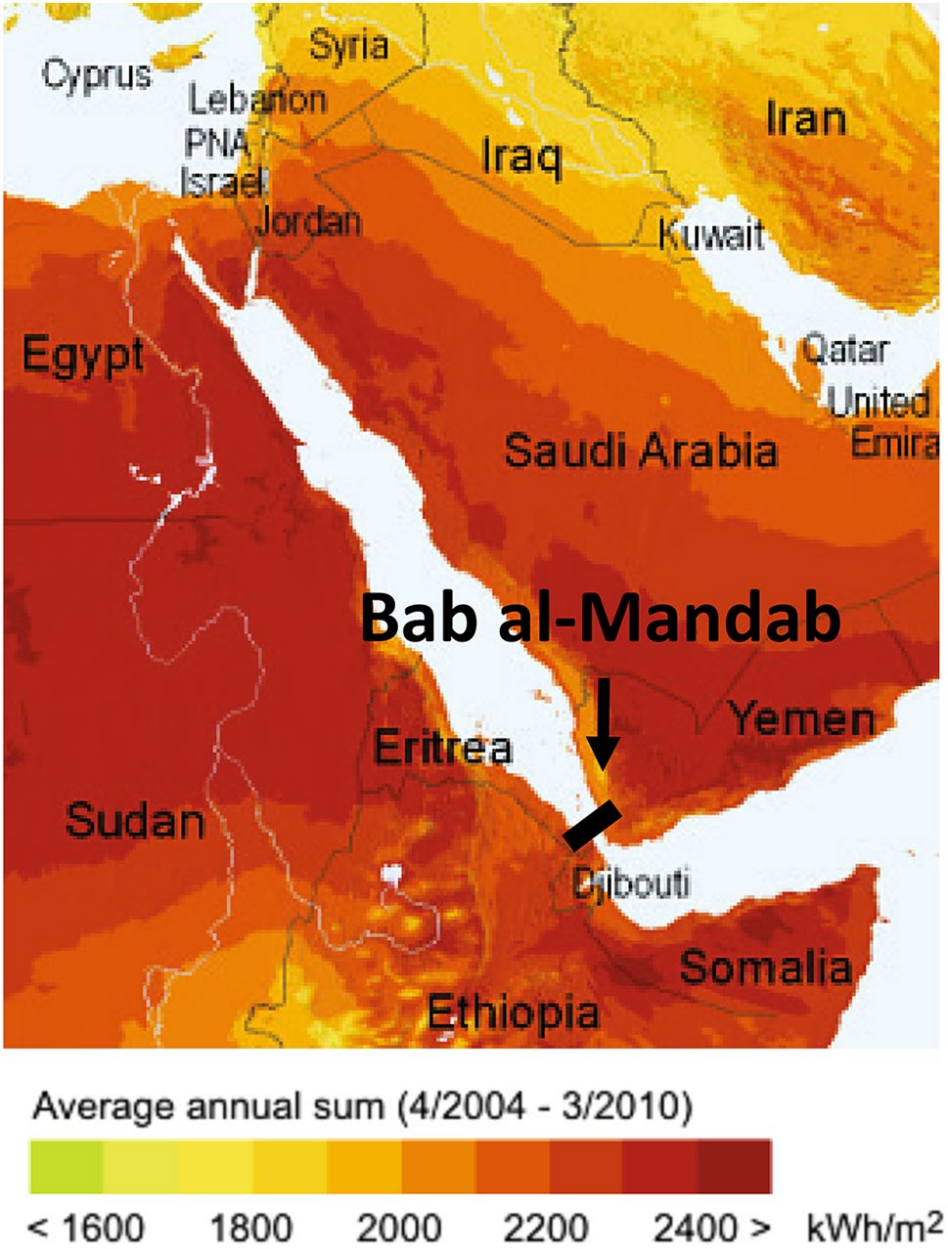
Solar irradiation map of the Red Sea (Solargis 2016). The fast evaporating water of the Red Sea is almost exclusively replenished through the Bab al-Mandab strait. Damming the 130-km-long strait could be used for 1 TW of hydroelectricity

An example of high potential of hydro energy is the case of the Red Sea. It is unique because it is not fed by any significant freshwater source. It has a surface area of 438,000 km^2^, is located in an extremely arid region, and has a very high evaporation rate (Ben-Sasson et al. 2009). Its evaporation is almost exclusively compensated by water replenishment from the Indian Ocean through the Bab al-Mandab strait. This unique situation could be considered for hydroelectricity (Fig. 1). If Bab al-Mandab were to be dammed, the Red Sea level would drop by 8 m in a period of 4 years. The resulting gravitational potential energy could generate electricity while simultaneously being sustained by continuous evaporation. This gigantic endeavor would result in approximately 1 TW of clean, sustainable, and non-variable electricity. It would cover the entire energy demand in the Middle East and could supply Europe with complementary energy for a variable renewable energy system based on solar panels and windmills. However, it would cover only an additional 5.1% of the global energy demand met by fossil fuels in 2013. This approach of using solar energy is sustainable, clean, non-variable, and economically viable, but the solar-to-gravitational potential energy conversion efficiency is less than 0.01%, while the density of the gravitational potential energy is 1 MWh per 46,000 m^3^ of water. Therefore, the available solar energy is minimally exploited and a tremendous scale of water is transported for a relatively low electricity output. Furthermore, a tremendous initial damage to the established ecosystem would be expected. The relatively low energy output of this unique example of large-scale clean, sustainable, and non-variable energy production stresses the gravity of the world energy problem.

## Sustainable energy economy

### Solar energy—the only energy source capable of eradicating energy-related GHG emissions

Solar energy is the most abundant energy source on our planet. The energy irradiated by the sun on the world’s land surface is 23,000 TWyear (IEA 2015c), which translates into an extremely high energy density. The energy irradiated on a surface of 95,000 km^2^ would cover the entire energy demand covered by fossil fuels in 2013. In contrast, wind energy has a relatively low energy density. The global wind energy potential is estimated at only 72 TWyear and is relatively evenly distributed over the globe (Archer 2005). Therefore, using wind energy to substitute fossil fuels and ensure prolonged energy security would require a non-feasible deployment of wind farms. Any other available renewable energy source might be relevant to reduce GHG in the bridge scenario. However, their theoretical global potential is way too low even to be considered as a solution to our energy and climate problem (IEA 2015c). Solar energy is therefore the only available energy source with a real potential for eradicating GHG emissions while ensuring sustained energy security.

Photovoltaics (PV) have a high energy conversion efficiency. Systems exist with an energy conversion efficiency as high as 43% (Steiner et al. 2016), although commercially available panels have a conversion efficiency average of 12–18%. The most irradiated areas in the world are the Atacama in South America, Sahara in Africa, and Great Sandy in Australia (Solargis 2016). They receive an annual irradiance of 0.27 TWyear per 1000 km^2^. If PV farms with a conversion efficiency of 15% were to be built in these regions only, an area of 495,000 km^2^ would be sufficient to cover the entire global energy demand projected for 2030 in the bridge scenario. However, the energy output of PV panels is electricity, and its storage and transportation are very limited. PV electricity is required to be consumed immediately and locally. Looking at the Czech Republic as an example for a temperate country, we find an irradiance of 0.13 TWyear per 1000 km^2^. It would require PV on an area of 14,700 km^2^ to cover its entire energy demand in 2012 (World Bank 2015). This area is 19% of the country’s total, which might not be viable when considering that 55% of the land is used for agriculture and another 34% is covered by forest (Trading Economics 2016). The Czech Republic as well as most of Europe would have to import a great amount of their energy needs from southern Spain and Turkey with considerable energy losses during transmission and a correspondingly elevated price. They might compensate for the deficit partially with wind energy; however, a more critical issue is the variable nature of both solar and wind energy. Solar energy is available only during daytime, and the daily irradiance depends on season and climate. Therefore, the very limited capability of storing electricity is the major problem for an energy system based on PVs. An additional concern about a large-scale PV energy production system is the high price, toxicity, and non-recyclability of rare metals required for the production of PV panels (Kleijn and van der Voet 2010; Elshkaki and Graedel 2015).

Biofuels are energy carriers resulting from solar energy capture by photosynthesis. A major advantage of biofuels over photovoltaic electricity is their flexibility as energy carriers. They can be stored and transported like fossil fuels while using the existing infrastructure. There are three generations of biofuels. The first generation consists of using crops with high sugar or oil content (e.g., corn, sugarcane, soybean) to produce bioethanol or biodiesel. This production is deployed at high scale and covered about 7% of the world’s energy consumption in 2013 (Ho et al. 2014). However, its production consumes arable land and causes an increase in food prices, endangering food security. The second generation of fossil fuel uses agricultural and forestry biomass waste for biofuel production. These fuels are generated without competing with food for arable land. In 2013, such biofuels covered about 3% of the global energy demand (Ho et al. 2014). An increase in the production of second-generation biofuels is much desired. However, its deployment is expected to be quite limited due to very low energy conversion efficiencies, technical difficulties, and the limitation of their production to the available waste. A third generation of biofuels would rely on microalgae (Voloshin et al. 2016; Rodionova et al. 2016). Microalgae are robust unicellular organisms that can be grown in seawater or wastewater that can be installed on land unsuitable for agriculture, and they have higher energy conversion efficiency than the other two categories of fuels. They represent a viable solution to the problems posed by first- and second-generation fuels. However, the use of biofuels is far from being carbon neutral. In addition to the CO_2_ emitted by the use of biofuels, a considerable quantity of emissions is caused during their production and isolation, which is not compensated by CO_2_ fixation.

### H_2_ from H_2_0—a clean, sustainable, and flexible energy carrier

Hydrogen is the most abundant element in our universe. On Earth, it is found to be mainly associated with oxygen in the form of water but also in other compounds such as organic matter and fossil fuels. With an external input of energy, hydrogen can be extracted from these compounds to generate molecular hydrogen. Molecular hydrogen is an energy carrier that recombines easily with oxygen. This reaction results in the release of energy and water. If molecular hydrogen is extracted from water, the byproducts of its production and consumption are almost fully recycled. Therefore, molecular hydrogen has a high potential as a clean and sustainable energy carrier. During the 1970s, a rise in oil prices brought up the concern that the use of fossil fuels is bound not only to environmental damage but also to future economic damage. Reduced availability caused by declining reserves or political instability in countries holding major reserves can cause immediate damage to the world economy and development. In view of this, the hydrogen economy was proposed. In such an economy, hydrogen would replace fossil fuels and take over the role of main energy currency (Veziroğlu and Şahi˙n 2008). The specific energy released by combustion is higher than that released by the combustion of any fossil fuel. Hydrogen can be transported over large distances by hydrogen trucks, tankers, and pipelines and be stored for long periods in mass quantities. Hydrogen can also be compressed and used on board vehicles. This high flexibility makes hydrogen an excellent substitute for fossil fuels.

To replace fossil fuel use by a hydrogen economy, an entire infrastructure for hydrogen use needs to be established. This infrastructure includes hydrogen production, storage, transmission, distribution, and conversion. In 2013, global hydrogen usage amounted to a corresponding energy of 0.23 TWyear (IEA 2015d). This amount could cover the entire energy demand met by nuclear energy in 2013. However, most of the hydrogen produced is used by the chemical industry and not the energy sector. About 90% of this hydrogen is extracted from fossil fuels using extreme heat and pressure as input energy (Krishna 2013). The remaining carbon is released in the form of CO_2_. Large-scale energy storage demonstration projects are being launched, planned, or announced, with a high concentration of activity in Germany. In Japan, a hydrogen supply chain is being established to import Australian hydrogen to Japan by liquefied hydrogen carriers similar to liquid natural gas carriers. A pilot hydrogen import hub is being built and is expected to start operation in 2020. Considerable experience in transmitting hydrogen on land via pipeline already exists. In the United States, the existing hydrogen pipeline system amounts to some 2400 km; in Europe, almost 1600 km are already in place (Pacific Northwest National Laboratory 2015). Hydrogen can be converted to electricity and heat by fuel cells. The conversion efficiency of hydrogen to electricity of 35–50% is relatively low due to energy dissipation through heat. However, by using the heat in a co-generation system, a total energy conversion efficiency of 80–90% can be achieved. This efficiency level has been demonstrated on a large scale in Japan where more than 120,000 “Ene-farm” domestic fuel cell micro co-generation systems are already installed to provide domestic electricity and heat (NEDO 2014). In the transportation sector, fuel cell-powered vehicles (FCEVs) debuted commercially, with Toyota introducing its Mirai to the Japanese, German, and the US markets, while Hyundai will present its own FCEV soon. Advanced FCEVs have a range of 500 km with a full tank. Hydrogen fueling stations are being built in major Japanese, German, and the US cities.

The hydrogen economy is thus being developed and deployed, but to reach an economy of scale, high investment and governmental incentives will be required to drive a fast rollup. In the case of successful rollup, the IEA assessed that by 2030 hydrogen technology will be priced competitively with fossil fuel technology. However, hydrogen is mainly produced from fossil fuels, and the available hydrogen has an unclean footprint. Generation of hydrogen from fossil fuels will entail localized pollution, which can be mitigated by CO_2_ capture and sequestration and will power the development of the hydrogen economy infrastructure during the bridge scenario period. However, to have a fully sustainable and clean hydrogen economy, hydrogen needs to be produced from water with the input of a sustainable and clean primary energy source (e.g., solar energy).

### Solar to hydrogen energy

The only current commercially available method to use solar energy to extract hydrogen from water is by coupling PV to electrolysis. With an electrolysis efficiency of 70%, a total solar-to-hydrogen energy conversion efficiency of 8–13% can be achieved. From the point of view of the required land use, this approach could be a viable solution to a sustainable hydrogen economy. However, the high price of PV coupled to electrolysis and the use of expensive and polluting materials still remains an obstacle to reaching the required scale. An increase in efficiency could overcome this obstacle.

Using photonic energy to split water without having the intermediary step of electricity generation could result in a better solution. In this sense, much research in artificial photocatalytic water splitting is being performed (Abdelaziz et al. 2013; Eaton-Rye 2013; Ismail and Bahnemann 2014; Barber 2016; Acar et al. 2016). This technology consists of metallic catalysts that absorb light and create charge separation, which can be used as oxidizing and reducing power for water splitting and H_2_ production. However, many obstacles remain to be overcome. Most available catalysts absorb in the UV spectrum, while most of the solar irradiance is in the visible spectrum. The generated electron–hole caused by photonic energy recombines easily. The catalysts are quite unstable and are not usable for prolonged periods. Hydrogen production occurs in parallel to oxygen production, and the required rapid separation of these gases is hampered by technological barriers and high costs. In an attempt to overcome some of the obstacles, a system consisting of two different catalysts complemented by electron shuttle molecules was developed following a Z-scheme. This construct enables light absorption in the visible spectrum and limits charge recombination for improved electron transfer. The system follows an example from nature, namely the photosynthetic electron transport (PET) chain from oxygenic photosynthesis.

Photosynthesis is one of the most important life-sustaining reactions on our planet. It provides the energy required for the survival of all life forms and underlies the accumulation of fossil fuels (Taiz and Zeiger 2002). This complex molecular machinery evolved over the past 3 billion years for the efficient conversion of sunlight to chemical energy, which to date has not been matched by any human-made technologies (Nelson 2011, 2013). Most of the sunlight irradiated on our planet is in the visible spectrum. In plants and microalgae, oxygenic photosynthesis uses large antenna proteins with associated green pigments called chlorophylls to harvest light in most of the visible spectrum. The absorbed light is funneled with very high efficiency towards the reaction centers of the protein complexes PSII and PSI. This reaction drives the PET chain to extract electrons from water for CO_2_ fixation and biomass production.

Some microalgae express a highly active [FeFe]-hydrogenase which, coupled to the PET chain, can accept photosynthetic electrons from water for hydrogen production. This system has clear advantages over artificial photocatalytic water splitting. The quantum yield of electron transported per absorbed photon is close to 1. The system does not require rare and expensive metals. PSI is highly stable while the unstable reaction center of PSII is continuously repaired. A major remaining issue is the separation of oxygen and hydrogen production, especially given that the [FeFe]-hydrogenase is highly oxygen sensitive and irreversibly inactivated by it. Nevertheless, Melis et al. (2000) discovered that under sulfur starvation in the light, net oxygen production drops below zero and continuous anoxic hydrogen production can be observed. The reason is that the carbon fixation, starch storage, and O_2_ respiration machinery of the cell complements the PET chain in favor of anoxic hydrogen production. This highly sophisticated and flexible system is therefore of great interest for a clean and sustainable hydrogen economy.

## Photosynthetic hydrogen production

Photosynthesis takes place in the chloroplast and can be divided into two steps: the thylakoid reactions (or light reactions) and the carbon fixation reactions (Taiz and Zeiger 2002).

**Fig. 2:**
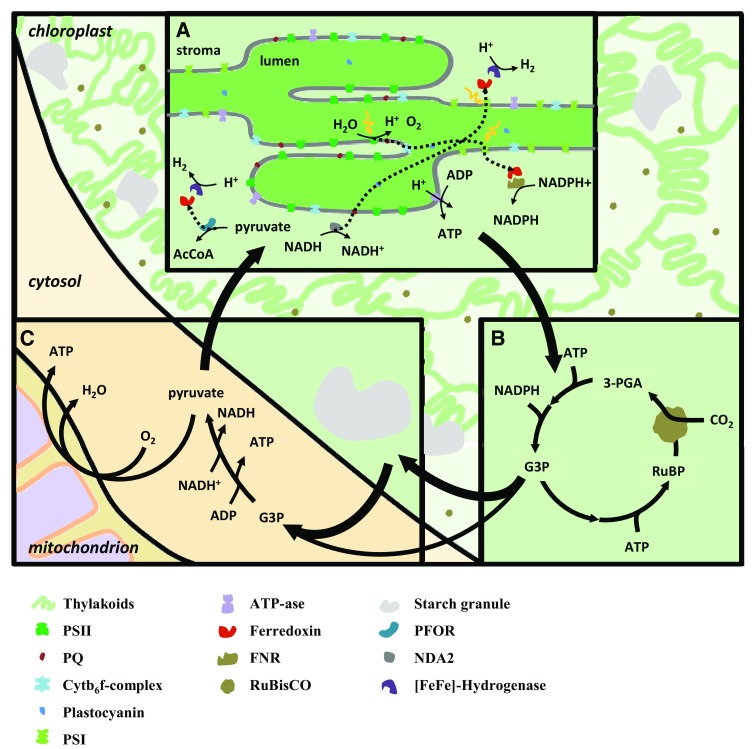
Indirect pathway for photosynthetic H_2_ production. The thylakoid reactions (*light* reactions) use light energy to oxidize H_2_O and generate ATP and NADPH (**a**). The carbon fixation reactions (Calvin–Benson cycle) use ATP and NADPH to fix CO_2_ and generate triose phosphates (G3P) (**b**). G3P can be stored as starch or transported to the cytosol and undergo glycolysis to yield ATP, NADH, and pyruvate (**c**). Pyruvate and NADH are used for light-independent and light-dependent (PSII-independent) H_2_ production, respectively (**a**). Pyruvate can also drive respiration in the mitochondrion for ATP synthesis (**c**). *Dotted arrows* indicate electron transfer

The light-harvesting antennae are embedded in the thylakoid membranes of the chloroplast and are associated with the protein complexes designated as Photosystem II (PSII) and Photosystem I (PSI). The reaction centers of PSII and PSI are responsible for converting light energy into redox potential, which drives the PET chain. The PET chain oxidizes water in the lumen and transfers the obtained electrons through PSII, plastoquinone (PQ), cytb6f complex, plastocyanin, and PSI to reduce ferredoxin in the stroma. The thylakoid reactions result in oxygen, ATP, and NADPH production by PSII, ATP-synthase, and ferredoxin-NADP+-reductase (FNR), respectively (Fig. 2a). The [FeFe]-hydrogenase is also coupled to the PET chain and can accept electrons from ferredoxin for hydrogen production.

ATP and NADPH are energy carriers required by the Calvin–Benson cycle to fix carbon into organic molecules. CO_2_ is fixed into ribulose-1,5-bisphosphate (RuBP) by the carboxylase activity of ribulose-1,5-bisphosphate carboxylase/oxygenase (RuBisCO), resulting in two molecules of 3-phosphoglycerate (3-PGA). These molecules are reduced to triose phosphates (G3P) at the expense of energy in the form of ATP and NADPH. At the expense of additional ATP, one molecule of RuBP is regenerated from five molecules of G3P. The carbon fixation reactions use energy delivered by the thylakoid reactions to synthesize one molecule of G3P for every three cycles of CO_2_ assimilation (Fig. 2b).

G3P is an organic energy carrier that can undergo transformation in the chloroplast and be stored as starch. When required, starch deposits break down to release it. G3P can be transported out of the chloroplast and consumed by diverse metabolic pathways to fuel the immediate energy and biomass needs of the cell. In the cytosol, glycolysis converts G3P to pyruvate and makes energy available in the form of ATP and NADH (Fig. 2c). Pyruvate can be further metabolized by the citric acid cycle in the mitochondrion to drive oxidative phosphorylation. This uses the reducing equivalents delivered by pyruvate to reduce O_2_ to H_2_O and drive ATP synthesis. Cytosolic glycolysis and mitochondrial respiration result in the release of photosynthetic energy stored in G3P and sequestrates oxygen into water.

This sophisticated energy absorption, conversion, and storage machinery evolved to supply the cell with the required energy for proliferation and maintenance while being exposed to a series of environmental changes (e.g., light intensity and air composition). To do so, this machinery is complemented by a complex regulation system to orchestrate its activity. It is crucial to consider this regulation when exploiting photosynthesis for enhanced hydrogen production. Oxygenic biophotolytic hydrogen production can occur through the direct pathway (PSII dependent) or the indirect pathway (PSII independent) (Burgess et al. 2011).

The direct pathway requires PSII activity and directs electrons extracted from water to immediate hydrogen production. The indirect pathway consists of two steps. In the first step, energy is captured by photosynthesis and stored as starch. In the second step, stored starch is glycolysed, and the resulting NADH and pyruvate are used for PSII-independent hydrogen production (Fig. 2). NADH can feed electrons into the PQ via a type II NADH dehydrogenase (NDA2) and drive the PET chain for light-dependent (PSII-independent) hydrogen production. In contrast, pyruvate is used by the pyruvate:ferredoxin oxidoreductase (PFOR) to reduce ferredoxin and drive light-independent hydrogen production.

Using this mechanism for mass hydrogen production will require a system that provides anoxic hydrogen production, high energy conversion efficiency (i.e., 5%), and efficient upscale.

### Anoxic hydrogen production

The PSII-dependent pathway has the highest theoretical energy conversion efficiency. However, it cannot be fully exploited since it entails simultaneous hydrogen and oxygen production. In contrast, the PSII-independent pathway does not. To exploit the PSII-independent pathway for temporally separated oxygen and hydrogen production, PSII activity needs to be controlled. In a first stage, full PSII activity harvests solar energy, and the carbon fixation reaction stores it in the form of starch, lipid, or other biomass. In a second phase, PSII activity is turned off, O_2_ production stops, and hydrogen production takes place by consuming previously stored biomass. Using starch for temporary energy storage results in reduced energy conversion efficiency compared to the direct pathway. However, the indirect pathway does not compromise anoxic hydrogen production. The sulfur starvation protocol relies on both pathways. It consists of reducing PSII activity. The subunit D1 of PSII is frequently damaged by photosynthetic activity and replaced by newly synthesized protein. Sulfur is essential for protein synthesis. Therefore, the activity of PSII is gradually decreased down to 10% upon sulfur shortage. The low PSII activity accompanied by an unchanged cell respiration results in net O_2_ consumption. Additionally, a rapid increase of starch and the concomitant degradation of RuBisCO are observed during the initial stage of sulfur starvation. This is followed by gradual starch degradation and hydrogen production by the PSII-dependent and PSII-independent pathways simultaneously (Zhang et al. 2002). Hydrogen productivity and the contribution from each pathway depend on light intensity. Laurinavichene et al. (2004) reported the highest productivity at 30–40 µE m^−2^ s^−1^. A reduced productivity at higher light intensities has been related to higher PSII activity. Most sulfur starvation studies include acetate as an exogenous organic carbon source. In addition, pyruvate can drive oxygen respiration. Therefore, it plays an important role in maintaining anoxic conditions (Fouchard et al. 2005; Degrenne et al. 2010). The addition of an exogenous carbon source implies exogenous energy input and results in a non-sustainable hydrogen production system. Acetate-free hydrogen production under sulfur starvation has been reported as well (Tsygankov et al. 2006; Tolstygina et al. 2009). In order to accumulate sufficient starch and reach anoxic conditions for hydrogen production, cultures needed sufficient CO_2_, a specific light regime, and pH control. Photoautotrophic hydrogen production needs to be further investigated and is crucial for sustainable hydrogen production. The sulfur starvation protocol is widely used to study hydrogen production in microalgae, but it entails great impracticality in an upscale system because it would require the tedious transfer of cells from sulfur-replete to sulfur-depleted media. Additionally, no wastewater could be used for this purpose due to naturally occurring sulfur, which would even further increase the costs and reduce the sustainability of a hydrogen production plant based on the sulfur deprivation protocol.

Other nutrient deprivation methods like nitrogen, potassium, and phosphorus have been tested and resulted in hydrogen production (Philipps et al. 2012; Batyrova et al. 2012; Papazi et al. 2014). A drawback of nutrient deprivation is the eventual cell death. However, this can be avoided by repleting the starved cells with the missing nutrient. Another attempt to regulate PSII activity involved the use of an inducible promoter (Surzycki et al. 2007). A copper-inducible promoter was used to regulate the accumulation of the PSII subunit D2; however, this promoter proved ineffective because it was also activated by anoxic conditions. Such methods may improve the viability regarding the use of water and cell death. Regarding the tedious transfer of cells from different media, studies have proposed immobilizing cells on different materials (Laurinavichene et al. 2006; Kosourov and Seibert 2009), which would indeed greatly facilitate the transfer of cells. Furthermore, an increase in yield was observed in these systems due to a higher cell density and better light penetration. However, none of these systems have been tested yet at a large scale, and their feasibility needs to be validated. In a different approach, our lab proposed using temperature to control PSII activity (Mazor et al. 2012; Bayro-Kaiser and Nelson 2016). For this purpose, randomly generated mutants were screened for PSII temperature sensitivity. These mutants revealed the reduction of PSII activity upon incubation at higher temperature. When oxygen production levels drop below respiration levels, cells can maintain anoxic conditions and produce hydrogen at similar rates attained under the sulfur starvation protocol. Using differential temperature as a controlled PSII activity switch seems quite feasible on a higher scale. In mutant TSP4, PSII is totally degraded at high temperature. Therefore, maintaining anoxia does not require acetate or a strict CO_2_, light, and pH control. However, this mutant relies only on the PSII-independent pathway for hydrogen production. An increase in the efficiency of this pathway will be required to compensate for the inactive PSII-dependent pathway.

Alternating oxygenic photosynthesis and anoxic hydrogen production is a very promising approach for sustainable and clean hydrogen production. However, for mass hydrogen production, minimal energy conversion efficiency is required to reach economic viability. The theoretical maximal solar-to-hydrogen energy conversion efficiency is 12–14% (Eroglu and Melis 2016), and it has been estimated that an efficiency of 5% will be sufficient for an economically viable system if it is coupled to the co-production of high-value products (Kruse et al. 2005b). Current efficiencies of sulfur-starved algae may reach levels up to 1–2% under laboratory conditions, but Torzillo et al. (2015) reported that a significant reduction in energy conversion efficiency is observed in large-scale bioreactors. An efficiency of 0.2% has been reported in a 110-L photo-bioreactor (PBR). Improvement of both the biological system in the laboratory and the environmental conditions in the upscale system is crucial to attain a minimal 5% of solar-to-hydrogen energy conversion efficiency.

### Improving energy conversion efficiency in the laboratory

Microalgae do not require the entire extent of energy delivered by solar light to perform photosynthesis and produce biomass. Therefore, the light-harvesting systems evolved to dissipate most of the incident light in the form of fluorescence and heat. Truncated antenna in *Chlamydomonas reinhardtii* TLA mutants demonstrated that smaller antenna resulted in less dissipated light energy and a higher hydrogen production yield per culture (Tetali et al. 2007; Kosourov et al. 2011; Perrine et al. 2012). This higher yield is not a result of higher single-cell photochemistry. However, light that is not used for photochemistry by cells on the culture’s surface is not dissipated as fluorescence or heat. Instead, it penetrates the microalga culture and enables cells deep inside the culture to receive sufficient light for photochemistry.

Under high light intensities, reaction centers may saturate and the lifetime of the excited state of chlorophylls may be prolonged. The result may be an intersystem crossing of the excited chlorophylls to the triplet state. This species reacts with oxygen, yielding reactive oxygen species (ROS), which cause photoinhibition as well as further cell damage. To reduce the risk of ROS production, protective non-photochemical quenching (NPQ) mechanisms are activated under high light intensities. Lumen acidification due to high photochemical activity induces the expression of light-harvesting complex stress-related (LHCSR) proteins in microalgae (Bonente et al. 2011). These proteins absorb the excess energy and dissipate it rapidly in the form of heat and fluorescence. The *C. reinhardtii* mutant npq4 lacks two of the three LHCSR proteins. This mutant has a lower NPQ, higher biomass production, and the same amount of ROS accumulation compared to the wild type (Berteotti et al. 2016). However, a mutant lacking all three LHCSR proteins accumulates higher amounts of ROS and cannot grow at high light intensities. The authors propose that NPQ is an overprotective mechanism that favors reducing the risk of photodamage over higher productivity. Another important mechanism for NPQ is state transition. A highly reduced plastoquinone due to high PSII activity induces the phosphorylation of the light-harvesting system of PSII, resulting in their migration towards PSI (i.e., state 2) (Lemeille et al. 2010). This mechanism modulates photosynthetic electron flow by redistributing light-harvesting complexes between PSII and PSI. The *C. reinhardtii* mutant stm6 is locked permanently in state 1, favoring linear electron flow over cyclic electron flow (Kruse et al. 2005a). As a result, this mutant has a higher starch accumulation and a higher hydrogen production rate compared to the wild type.

The Calvin–Benson cycle is the main consumer of the reducing power generated by the PET chain, while it is responsible for carbon fixation and starch production. Downregulation of the carbon-fixing protein RuBisCO has been proposed to direct photosynthetic electrons towards hydrogen production (Marín-Navarro et al. 2010). The *C. reinhardtii* mutant CC-2803 lacks RuBisCO activity and has a higher hydrogen production rate under sulfur starvation compared to the wild type (Hemschemeier et al. 2007). However, this mutant can grow only under dim light, and hydrogen production relies only on residual PSII activity. Therefore, the indirect pathway cannot be exploited to further increase hydrogen production. In another study, *C. reinhardtii* mutants with reduced RuBisCO levels, activity, and stability were engineered (Pinto et al. 2013). The low level of RuBisCO present in these mutants is increased significantly during the initial stage of sulfur deprivation and eventually reduced drastically. This change enables these mutants to initially produce and accumulate starch, while during hydrogen production no RuBisCO activity consumes electrons. The mutant Y67A exhibited a 10- to 15-fold higher hydrogen production compared to the wild type. Ideally, RuBisCO activity should be as high as possible for starch production and downregulated completely for hydrogen production. In this sense, the *C. reinhardtii* mutant 68-4PP might be of use. This mutant exhibits a temperature-sensitive RuBisCO (Chen et al. 1993).

RuBisCO exhibits carboxylase activity as well as oxygenase activity. Although its specificity towards CO_2_ is 88 times higher than that towards O_2_, the usually low CO_2_ concentration in air results in a 25% oxygenase activity and a respective energy loss. Improved carboxylase activity would improve energy conversion efficiency and can be achieved by increasing the CO_2_ concentration. By adaptive laboratory evolution, *Chlorella* sp. strains with high CO_2_ tolerance have been generated (Li et al. 2015a). Strain AE10 grows rapidly at 30% CO_2_ and produces almost three times more biomass than the parental strain.

Accumulation of high amounts of starch is critical for subsequent PSII-independent hydrogen production, a given fact under the sulfur deprivation protocol. Sulfur and other nutrient shortages induce stress responses in algae, which result in rapid and high starch accumulation (Melis et al. 2000; Philipps et al. 2012). This accumulation is a practical added value of using nutrient deprivation for hydrogen production, which might not be the case for the temperature-sensitive PSII approach. By forward genetics, the *C. reinhardtii* mutant std1 with high starch accumulation under nitrogen deprivation has been isolated (Schulz-Raffelt et al. 2016). Std1 lacks the dual-specificity tyrosine phosphorylation-regulated kinase (DYRK). The authors propose that DYRK is activated by nutrient shortage and is a negative regulator of the induced higher starch accumulation. Therefore, inactivation of DYRK results in an improved starch response to nutrient stress. Not many studies have been performed to increase starch biosynthesis, but many groups have pursued the inhibition of starch biosynthesis to increase lipid production (Li et al. 2010; Work et al. 2010; Johnson and Alric 2013). Lipid and starch biosyntheses are competing metabolic pathways. Therefore, they might be suggested to downregulate lipid production to increase starch storage.

Starch breakdown through glycolysis results in NADH and pyruvate production. NADH can reduce the PQ pool via NDA2 to drive the PET chain for light-dependent (PSII-independent) hydrogen production, while pyruvate can be consumed by PFOR to reduce ferredoxin and drive light-independent hydrogen production. Salt stress has been reported to induce starch breakdown in the salt-tolerant green algae *Dunaliella tertiolecta* and might be a practical tool to induce PSII-independent hydrogen production (Goyal 2007). Overexpression of NDA2 was achieved in the *C. reinhardtii* mutant CrNDA2 by genetic engineering. The Nda2 gene was integrated into the chloroplast genome under the control of the highly expressed psaA promoter (Baltz et al. 2014). Under sulfur starvation, this mutant exhibits a higher hydrogen production by the indirect pathway, while the efficiency of the direct pathway is unchanged compared to the wild type. Pyruvate resulting from starch glycolysis may drive light-independent hydrogen production via PFOR; however, many other metabolic pathways compete for pyruvate (e.g., lipid, lactate, acetate, formate, and ethanol production). These pathways are proposed as targets for metabolic engineering to increase light-independent hydrogen production (Doebbe et al. 2010). The *C. reinhardtii* PFL1 mutant lacks the PFL enzyme, which metabolizes pyruvate to formate (Philipps et al. 2011). This mutant exhibits no formate production but does have higher lactate, ethanol, and light-independent hydrogen production; however, light-dependent hydrogen yield is reduced. The authors suggest that this decrease results from a lower expression of [FeFe]-hydrogenase in the mutant compared to the wild type.

During the hydrogen production stage, the goal is to funnel the electrons from the PET chain towards the hydrogenase instead of their being consumed for NADPH production or by other ferredoxin electron acceptors. One approach previously mentioned would be to downregulate the main photosynthetic electron sink (i.e., the Calvin–Benson cycle). In another very clever approach, a ferredoxin–hydrogenase fusion protein was engineered and tested in vivo (Eilenberg et al. 2016). A gene encoding both ferredoxin and [FeFe]-hydrogenase with a [Gly4-Ser]3 linker in between was transformed into the nuclei of a mutant lacking the native [FeFe]-hydrogenase. It has been demonstrated that this mutant produces 4.5 times more hydrogen than the wild type.

An increase in energy conversion efficiency was achieved by manipulating various partial photosynthetic reactions involved in hydrogen production. Integrating such alterations into one system should be tested to maximize energy conversion efficiency.

### Efficient upscale photosynthetic hydrogen production

When upscaling photosynthetic hydrogen production, two important considerations must be addressed. The first is the choice of organism, and the second is the choice of bioreactor.

*Chlamydomonas reinhardtii* is the most used organism for hydrogen production studies in the laboratory due to its quality as a model organism. However, it might not be the most suitable organism for upscaling. *C. reinhardtii* grows best under moderate conditions (i.e., temperature, salinity, pH). Under these conditions, algal cultures are very susceptible to contaminating organisms, which reduce productivity dramatically. The sterility conditions that are possible in the laboratory cannot be assured in an upscale system without a tremendous economic burden. Maintaining a large-scale monoculture over longer periods is a challenge and crucial for efficient mass hydrogen production. Shifting the growth conditions to extreme conditions reduces susceptibility to contaminants. Some extreme conditions might even be required or beneficial for improved hydrogen production. High light irradiance and CO_2_ concentrations may be exploited for improved energy conversion efficiencies, while high salinity will enable the use of seawater for culturing. Under high light irradiance, the temperature is generally high as well. High temperature may be advantageous for the temperature-sensitive PSII protocol. There is a range of extremophile microalgae to be considered (Table 1). They grow at high temperature, light irradiance, salinity, and CO_2_ concentrations (Varshney et al. 2015). Their relevance for mass hydrogen production needs to be investigated. Substantial hydrogen production in seawater by marine microalgae under nutrient deprivation has been already reported (Batyrova et al. 2015; Li et al. 2015b). The chosen extremophile will have to be amenable to transformation to allow for genetic engineering of the photosynthetic apparatus for hydrogen production. Among the presented extremophiles, only *Dunaliella salina* has been reported to be transformed successfully (Tan et al. 2005; Feng et al. 2009). For the other organisms, transformation methods need to be tested and developed. On the other hand, past limitations for directed genetic engineering may be circumvented with the recent development of the Cas9-CRISPR technology (Barrangou and Doudna 2016).

**Table 1:** Extremophile green microalgae

Organism	Tolerated extreme conditions	Application	References
*Chlorella sorokiniana* UTEX 2805	High temperature (40 °C) and light intensity (2500 µE m^−2^ s^−1^)	Wastewater treatment	(de-Bashan et al. 2008)
*Desmodesmus* sp. F51, F2 and F18	High temperature (45 °C)	High lipid content and lutein production	(Pan et al. 2011; Xie et al. 2013)
*Chlorella kessleri* and *Scenedesmus obliquus*	High temperature (50 °C) and CO_2_ level (18%)		(de Morais and Costa 2007)
*Chlorella* sp. T-1, K35 and ZY-1	High temperature (40 °C) and CO_2_ level (70–100%)		(Hanagata et al. 1992; Maeda et al. 1995; Yue and Chen 2005)
*Dunaliella salina, bardawil*, and *tertiolecta*	High salinity (35%) and light intensity	b-carotene production	(Borowitzka and Siva 2007; Hosseini Tafreshi and Shariati 2009)
*Chlorella ohadii*	High light intensity (3500 µE m^−2^ s^−1^)		(Treves et al. 2013)

There are two distinct methods of mass microalga cultivation: the first is in open ponds and the second is in enclosed PBRs. Open ponds may be relevant only for the biomass production stage; the hydrogen production stage will require an enclosed PBR. The great advantage of open ponds is the low economic cost, but they offer little control over culturing conditions and result in low productivity. Biomass productivity has been reported to be around 0.2 g l^−1^ d^−1^ for different organisms in open ponds. *Dunaliella salina* is grown successfully in open ponds for b-carotene production. Biomass productivity in enclosed bioreactors has been reported to reach up to 2.4 g l^−1^ d^−1^. A series of different enclosed PBRs for medium- to large-scale applications are available and present different advantages (e.g., vertical column, tubular, flat panel) (Burgess et al. 2011; Adessi and De Philippis 2014). A first attempt of hydrogen production in an outdoor upscale PBR has been made in 2012 (Scoma et al. 2012). In the 50-l horizontal tube PBR, a decreased hydrogen output of 80% compared to laboratory conditions was observed. The authors attributed the reduced productivity mainly to different illumination patterns and mixing time. Shortly after, a 110-l horizontal tubular PBR immersed in a light-scattering nanoparticle suspension has been used for hydrogen production (Giannelli and Torzillo 2012). The enhanced light dilution resulted in two times better energy conversion efficiency compared to the previously reported PBR. However, many improvements are still required for reaching maturity. Critical aspects are shearing by mixing, light dilution, H_2_ escape, and economic costs (Skjånes et al. 2016).

## Prospects

Solar energy is the only energy source with the potential to fully replace fossil fuels, while hydrogen is a crucial energy carrier for ensuring energy availability across the globe. In order to provide a substantial addition of renewable energy and contribute effectively to the reduction of GHGs, solar-powered hydrogen production will have to comply with the parameters outlined by the example of hydroelectricity generation at the Red sea. The PSII-independent pathway of oxygenic photosynthetic hydrogen production is a promising option and we believe that a system based on a temperature-sensitive PSII mutant may comply with the requirements. It harvests solar energy for water splitting and evolves oxygen and hydrogen separately. The responsible machinery in the cell is complemented by a self-replication and auto-repair machinery, while the water source may be sea water. Reaching the required energy conversion efficiency of 5% is within the boundaries of thermodynamics and economic feasibility. A vast series of studies has already reported improved hydrogen yield by manipulating the photosynthetic machinery at different stages. These achievements may be engineered into a suitable extremophilic organism by the Cas9-CRISPR technology. Attempts at upscale systems are being made, while difficulties are being identified and addressed. Microalgal cells could be exploited for hydrogen production over five temperature cycles and the residual organic matter could feed an enormous number of livestock. This process yields real negative CO_2_ utilizable energy with minimal collateral pollution damage. We suggest that a process of integrating these methods into one system to achieve real sustainable energy conversion is already justified. This approach may lead to a mature technology for industrial deployment.
